# Sampling for Global Epidemic Models and the Topology of an International Airport Network

**DOI:** 10.1371/journal.pone.0003154

**Published:** 2008-09-08

**Authors:** Georgiy Bobashev, Robert J. Morris, D. Michael Goedecke

**Affiliations:** RTI International, Research Triangle Park, North Carolina, United States of America; University of Nottingham, United Kingdom

## Abstract

Mathematical models that describe the global spread of infectious diseases such as influenza, severe acute respiratory syndrome (SARS), and tuberculosis (TB) often consider a sample of international airports as a network supporting disease spread. However, there is no consensus on how many cities should be selected or on how to select those cities. Using airport flight data that commercial airlines reported to the Official Airline Guide (OAG) in 2000, we have examined the network characteristics of network samples obtained under different selection rules. In addition, we have examined different size samples based on largest flight volume and largest metropolitan populations. We have shown that although the bias in network characteristics increases with the reduction of the sample size, a relatively small number of areas that includes the largest airports, the largest cities, the most-connected cities, and the most central cities is enough to describe the dynamics of the global spread of influenza. The analysis suggests that a relatively small number of cities (around 200 or 300 out of almost 3000) can capture enough network information to adequately describe the global spread of a disease such as influenza. Weak traffic flows between small airports can contribute to noise and mask other means of spread such as the ground transportation.

## Introduction

Airline networks provide fast transportation every day for goods and people; however, these connections also provide pathways for the spread of diseases [1]. Recent real and hypothetical threats have increased interest about the role airline transportation has in the spread of HIV, severe acute respiratory syndrome (SARS), pandemic influenza, and drug-resistant tuberculosis (TB) [2]–[7]. Nevertheless, the role that airline transportation has on infectious disease epidemiology is not yet completely understood. Global cross-continental disease transmission is more likely by airline travel than by other means of travel, such as by boat. Additionally, air travel is the main form of transportation to remote areas such as small towns in Alaska, Siberia, and certain islands in the Pacific [1]. In these remote areas, public health interventions follow the same airline transportation routes as the spread of disease, meaning these routes to remote areas cannot be ignored. On the other hand, local transportation patterns might be more critical for the continental spread of influenza, and national grounding of air transport is believed to have little impact on fast disease propagation [8].

In their study, Guimerà et al. [1] emphasized the importance of understanding the connectedness of the world's populated areas. They showed that the most-connected cities are not necessarily the largest but play a critical role, not only for economic and cultural purposes, but also for global public health. Therefore, the role of both large and small airlines in providing community connections needs to be better understood. For example, researchers of global disease spread base their models on different numbers of airports, ranging from 52 to the entire sample of 3100 connected cities [2]–[4], [9], [10]. Relying on either extreme might lead to flawed results. On one hand, having too few cities could lead to researchers' missing important pathways that could be critical in disease spread. Samples from networks have been shown to lose many important properties of the entire network, such as scale-free degree distribution, and lead to bias in the main network measures [11]–[14], which might bias the patterns of disease transmission. On the other hand, using the entire sample of 3100 air transport connected cities leads to connectedness bias, because two cities can be weakly connected by air travel but heavily connected by ground transport. Finally, as was shown by Guimerà et al. [1], some of the most-connected cities are not necessarily the largest and, conversely, some of the largest cities (and thus very important for their role in epidemics) might not be the most connected.

Thus, in the present research we address two questions related to the network characteristics of disease spread on the samples of cities connected by air travel:

**(Q1)** *How does sampling affect the main network characteristics of the entire network?*

Although the small samples (representing major cities and continents) can produce strongly biased network characteristics, they might still be successfully used in adequate representation of global disease transmission. Thus, we ask the second question:

**(Q2)** *Can small samples capture enough network information to adequately describe the global spread of a disease such as influenza, and if so, then how should researchers select those samples?*

In this paper, we describe the data and sample selection rules, we describe network characteristics of the entire network and of the samples, we provide an analysis of disease spread on sampled networks, and, finally, we discuss the results and practical implications as they relate to the two research questions.

## Methods

### Data

Following Guimerà et al. [1] and Epstein et al. [4], we used flight information that commercial airlines reported to the Official Airline Guide (OAG) during the first week of November 2000. Cooper et al. [3] and Colizza et al. [2] report using similar data sets from 2002 reports, and Hufnagel et al. [15] have also used similar data. The OAG estimates that 99% of all commercial airlines report their daily scheduled flight information to the OAG throughout the year. For each scheduled flight, the airlines report the number of seats on the plane as well as the cities of origin and destination for the flight.

Because of the epidemiological implications, the unit of analysis in our study was a city rather than an airport. Most of the cities represented in the data set correspond to a single airport. However, for some larger cities, OAG has aggregated data from multiple airports. For example, New York City includes data from John F. Kennedy International Airport, LaGuardia Airport, and Newark Liberty International Airport. As another example, Washington, DC includes data from Dulles International Airport and Ronald Reagan National Airport but not Baltimore/Washington International Airport. The full OAG data set contains 3883 airport codes; however, a number of codes refer to train stations or bus stops. Additionally, some small airports (<100 passengers/day) are located on small islands for which population size was not available, and some airports are disconnected (i.e., do not have flights) from the main connected component. In our analysis, we have included only those locations from the largest connected network component for which we could obtain information on both flight volume and population size, resulting in a list of 2904 locations. We will refer to this reduced list as the OAG list.

We also considered a cruder aggregation of U.S. cities into larger metropolitan areas using the U.S. Census Bureau definitions of Combined Statistical Areas. For example, we aggregated Washington, DC, and Baltimore into a single Washington listing. This additional level of aggregation was performed to create a list of cities matching the one used in Epstein et al. [4], and, therefore, we will refer to it as the Epstein list.

### Samples

From the OAG and Epstein lists of cities, we selected several samples so that we may compare their network structures and their effects on a model of global disease spread. The samples varied by city list (OAG list, Epstein list), sampling method (most populous cities, cities with the greatest airline seat traffic, cities from Rvachev and Longini [10], cities from Epstein et al. [4]), and sample size. Following Rvachev and Longini [10], Epstein et al. [4], Cooper et al. [3], Colizza et al. [2], and Hufnagel et al. [15], we have selected networks containing the 52, 155, and 500 largest connected city nodes. We have also used the entire network of 2904 nodes. These sample sizes are equivalent to sampling proportions of 0.018, 0.05, 0.17, and 1 (on a natural logarithmic scale these sample sizes correspond approximately to 4, 5, 6, and 8). For the sampling methods, we will refer to sampling the most populous cities as *population-based*, sampling the cities with the greatest airline seat traffic as *volume-based*, sampling the Rvachev and Longini [10] cities as *Rvachev-based*, and sampling the Epstein et al. [4] cities as *Epstein-based*. Our set of samples included a sample that matched, or at least closely approximated, the list of cities used in Rvachev and Longini [10] (Rvachev-based, n = 52), Epstein et al. [4] (Epstein-based, n = 155), Hufnagel et al. [15] (volume-based, n = 500), and Colizza et al. [2] (volume-based, n = 2904).

For each sample, we created an adjacency matrix ***A*** such that an element *a_ij_* is equal to 1 when a flight exists between cities *i* and *j* and is equal to 0 otherwise. We also created a weighted adjacency matrix ***W*** such that an element *w_ij_* is the average daily number of seats on flights between cities *i* and *j*.

## Results

### Regional Coverage for the Samples and Flight Volume

**Figure 1:**
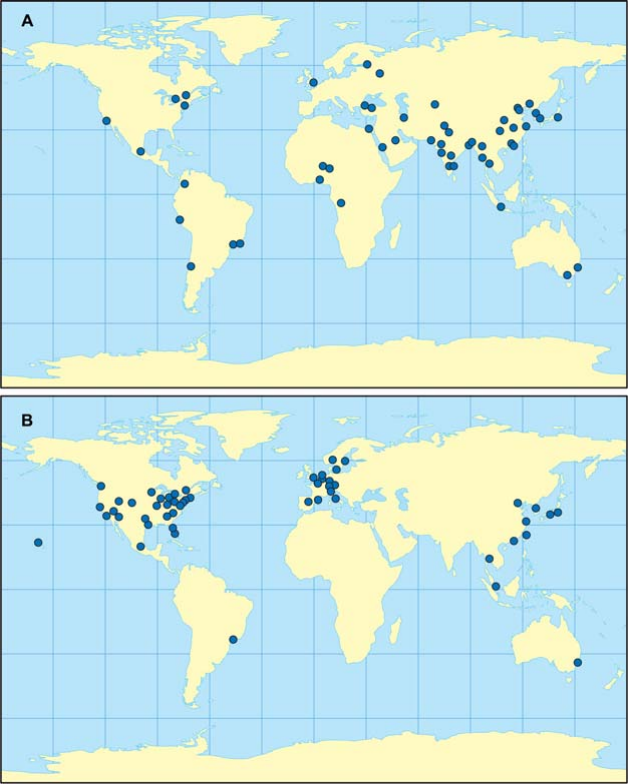
Map of the cities selected in (a) the population-based sample, n = 52 and (b) the volume-based sample, n = 52.

For global disease spread it is critical to include cities that cover all major regions. When the regions are defined broadly, a small sample size like 52 may or may not provide adequate coverage. In Table 1 and Figure 1, we see that the population-based, n = 52 sample provides reasonably good coverage if regions are defined as continents, while the volume-based, n = 52 sample includes no African cities and only one South American city. This result is not surprising since air traffic volume tends to reflect economic development, and so most of the highest-volume cities would be concentrated in developed parts of the world. Therefore, to obtain adequate regional coverage, volume-based samples typically need to be larger than population-based samples.

**Table 1:** Distribution of sampled cities across continents.

Region	52 cities, population-based	52 cities, volume-based	52 cities, Rvachev-based
Africa	5	0	6
Asia and Middle East	32	9	15
Europe	3	14	9
South America	5	1	7
North America	5	27	11
Australia and Oceania	2	1	4

When the regions of interest are small, such as the 101 regions recognized by the World Bank, one would expect numerous coverage gaps even with the population-based, n = 155 sample. Even some of the U.S. regions might not be adequately covered. For example, no Alaskan cities appear in any of the n = 155 samples. Many of these smaller regions rely on ground transportation for most local connections; in fact, more than 1000 of the 2904 cities in the OAG list have a total daily volume of fewer than 100 seats. Nevertheless, for places that are remote or are separated by country borders (such as Alaska) airline transport is critical for disease transmission.

In addition to regional coverage, global flight volume coverage may also be an important factor in modeling disease spread since the largest flight volumes may be more indicative of potential major disease routes. For example, a sample of 52 cities with the largest flight volume (which represents just 1.7% of the cities in the OAG list) accounts for about 40% of the total traffic. The coverage increases to about 75% for n = 155 and to about 90% for n = 500.

### Large-scale Structures of the Samples

**Figure 2:**
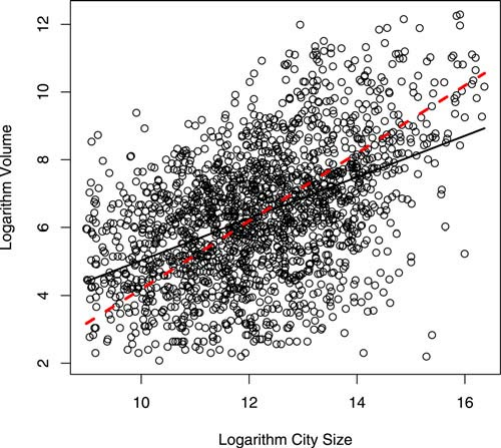
Log-log relation between the city and flight volume size in the data. Ordinary regression is represented with a solid black line, while an orthogonal regression is a dashed red. Orthogonal regression minimizes the orthogonal distance from the regression line as opposed to minimizing vertical distance in ordinary regression. The spread of the residuals is comparable to the range of the data indicating high variation of the flight volume for cities of the similar sizes. Orthogonal regression provides a useful reference line because it treats flight volume and city size as equal variables and could be viewed as the principle component capturing the essence of the relationship between the two variables. At the same time ordinary regression considers flight volume as a function of the city size. The lower slope in the regression line compared to the orthogonal regression indicates that there are many large cities that have a small flight volume as well as small cities with high flight volume.

A simple correlation analysis shows that city sizes and flight volumes are not strongly correlated to each other (Figure 2). The lower slope in the regression line compared to the orthogonal regression indicates that there are many large cities that have a small flight volume. One example of this type of city would be Lagos, Nigeria. On the other end of the scale, there are small hub cities like Frankfurt, Germany, where the daily flight volume can be comparable with the city size. Additionally, the city size distribution is not as steep as the size distribution for the flight volumes. For example, the 155 largest flight volume cities cover about 75% of all flight volumes while the 155 largest city populations contain less than 50% of the total city populations.

**Figure 3 a–d.**
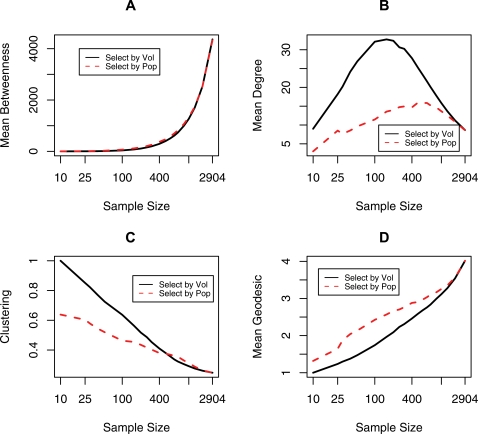
The relationships between network characteristics and sizes of network samples. Samples are selected based on flight volume (solid black line) and population (broken red line). Subplots correspond to the following network characteristics: (a) betweenness, (b) degree, (c) clustering, and (d) geodesic.

One would expect that as the sample size increases and includes more peripheral cities, the average shortest paths between the cities (the geodesics) would become longer and betweenness (the average number of geodesics passing through a node) would increase because a larger number of geodesics would pass through a node. These effects are in fact observed in the samples (Figures 3a and 3d). At the same time, the sample's average degree does not show a monotonic decrease. When the sample size is very small, the probability is low that cities connected to a selected city have also been selected into the sample. As more cities are added from the highly connected end of the city list, more connections are filled and the average degree increases. When smaller and less-connected cities are added, the average degree decreases (Figure 3b). The clustering coefficient (the probability that two cities connected to a third city are also connected directly to each other) monotonically decreases with increases in the sample size (Figure 3c). This outcome is associated with the results for the mean geodesic (i.e., for the small sample sizes almost all cities are directly connected to each other). As more cities are added these new cities are less likely to be clustered.

Although the trends are similar for the samples selected by volume and population size, there are differences in quantitative values. The peak of the mean degree for the volume-based samples is around 155 cities and the average degree in the samples is about 32. For the population-based samples, the peak corresponds to the largest degree of 500, and the average degree is around 17. This discrepancy reflects the fact that the largest cities are not necessarily the most connected nor do they have the largest flight volumes. The same argument applies to the clustering coefficient and the geodesic.

**Figure 4:**
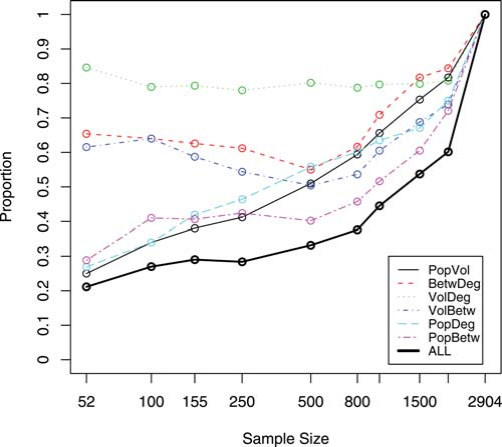
Proportion of cities sharing the top ranks between 2 or more characteristics. For each pair of network characteristics we consider 2 lists of cities ordered by each of the characteristics. The list size is shown on the horizontal axis. The proportion of cities shared by both lists is shown on the vertical axis. The minimum value of the shared proportion, 0, occurs when the lists have no city in common. The maximum value, 1, occurs when the two lists are identical. This is achieved only in the whole sample. For the 52 city lists, only 11 cities are among top 52 in all four characteristics.

As mentioned in Guimerà et al. [1] and Borgatti et al.[16], betweenness is a critical characteristic related to the bottlenecks in travel flows and disease transmission. Guimerà et al. [1] showed that the 25 most-connected cities are not necessarily the most central in terms of betweenness. Therefore, we evaluated the impact of the sample size and sampling type (population-based vs. volume-based). We have created lists of cities sorted by air travel volume, population size, city betweenness, and city degree. For each sample size, we calculated the percentage of cities common to two or more lists and present the results in Figure 4. For example, 80% of the 155 highest volume cities also appear among the 155 highest degree cities and vice versa.

**Figure 5:**
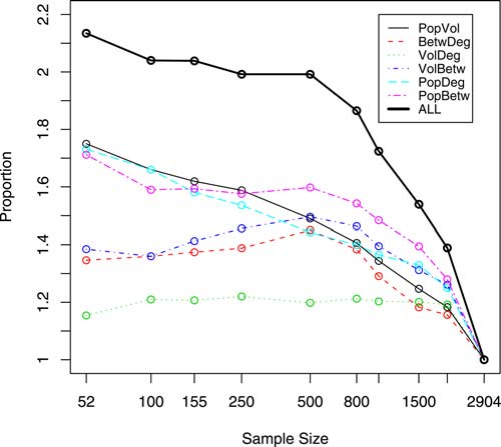
Inflation coefficient indicating the union of two ranking lists is larger than the size of a single list. For each pair of network characteristics, we consider two lists ordered by each of the characteristics. The single list size is shown on the horizontal axis. The inflation coefficient is shown on the vertical axis. The minimum value of the inflation coefficient, 1, occurs when the two lists are identical. This is achieved only in the whole sample. The maximum value of the inflation coefficient for the union of two categories, 2, occurs when the lists do not share any cities. For all four categories the maximum value of the inflation coefficient is 4. The size of the union of 52-city lists in all four categories is 2.2 times larger than the size of a single list, which translates into 114 cities.

The presented plots indicate that inclusiveness of volume, degree, and betweenness is fairly stable across the range of sample sizes. In particular, volume-based samples consistently contain more than 80% of the cities selected based on the highest degree and vice versa. The highest volume and highest betweenness samples share no less than 50%. In fact, the percentage of shared cities increases as the sample size gets larger than 1000. The highest betweenness and degree cities show a similar pattern. On the other hand, samples based on the population show little commonality (less than 30%) with samples based on other characteristics when the sample size is smaller than 100 cities. This percentage increases as the sample size increases. It is monotonic for population-volume and population-degree, but is not monotonic for population-betweenness. A sample as large as 1500 cities (about one-half of the total network) is needed to ensure that 50% of the cities appear on all four lists. If a researcher wants to include the cities that are within the top 100 of both population size and flight volume, he or she will need to have a sample size of about 165 cities (Figure 5). The multiplier generally decreases with increasing sample size; however, we observe that a nonlinear relationship between sample size and betweenness produces a bump in the three curves involving betweenness. Similar shapes, but the opposite directions, are observed in commonality plots.

### Disease Spread on the Sampled Networks

The Global Epidemic Model (GEM) has been developed as a tool for studying the global spread of influenza and consists of a number of coupled stochastic differential equations with parameters corresponding to the epidemiology and transmission of influenza [4]. The GEM is based on a number of previously published models, such as those by Rvachev and Longini [10] and Grais et al. [9], and is similar to the models developed by Colizza et al. [2] and Cooper et al. [3]. The GEM differs from other models mostly in the details of stochasticity (we used Poisson distribution for the numbers of infective contacts) and seasonality (we used a sinusoidal function with the amplitude depending on the latitude). The specific details about the model equations, table of parameters, and multi-lag travel matrix can be found in the supporting materials Text S1, Table S1, and Text S2, located in the supplementary material provided in Epstein et al. [4]. The requests for the code and information about the latest version of the model can be found at https://www.epimodels.org/midas/globalmodel.do. Thus, our model represents a larger group of robust, equation-based models describing the global spread of infectious disease. Using the GEM as a common modeling framework for all samples allows us to study the influence of sample size and sampling method on disease dynamics.

We tested the samples that include 52, 155, and 500 cities selected by population and volume, as well as the samples used in Rvachev and Longini [10] and Epstein et al. [4]. We have also included the entire network of 2904 cities and a sample of 204 cities composed of the union of cities in the top 100 in terms of flight volume, population size, betweenness, and degree.

Assuming that the flu epidemic started in Hong Kong on January 1, we used the GEM to calculate first passage times (FPT) to each city in the sample. The FPT to a particular city is defined as the number of days between the epidemic origination (the first day when 100 individuals in any single city became sick) and the moment when the number of infectious people in the destination city reaches or exceeds 1. The FPTs are presented in Table 2. Each number in the table is an average over 50 runs. The standard deviations varied between 3 and 6 days. We have highlighted the values that are significantly different from the estimates based on the entire census of the cities. A cell containing “–” indicates that the city was not selected for inclusion in that sample.

**Table 2:** First passage times (in days) to selected cities, assuming the epidemic originated on January 1 in Hong Kong.

Major Locations	52 Pop.	52 Vol.	52 Rvachev and Longini	155 Pop.	155 Vol.	155 Epstein	204 Combined	500 Pop.	500 Vol.	2904 Entire sample
Athens	–	–	–	–	64	65	65	64	64	64
Barcelona	–	72	–	–	71	71	70	70	70	70
Beijing	24	24	23	24	26	22	22	23	24	24
Bogotá	**125**	–	**114**	**111**	95	**102**	95	99	96	95
Cairo	**75**	–	69	69	69	68	68	68	66	68
Cape Town	–	–	58	**99**	57	58	59	58	59	58
Detroit	–	69	–	–	69	69	69	69	69	68
Fairbanks	–	–	–	–	–	–	–	–	–	112
Istanbul	–	–	–	49	49	49	49	49	49	50
Lagos	**104**	–	83	**93**	–	83	83	81	83	83
London	29	27	26	27	27	27	26	26	27	27
Los Angeles	27	29	28	28	27	29	28	28	30	26
Moscow	53	–	–	52	52	54	52	52	53	52
New Delhi	43	–	42	42	42	44	42	43	43	42
New York	**62**	55	**66**	57	53	59	54	56	54	54
Sao Paulo	79	77	79	77	74	76	75	75	74	75
Shanghai	20	21	20	20	20	19	20	20	21	21
St. Petersburg, Russia	**130**	–	–	**115**	98	94	92	95	93	94
Sydney	25	27	26	27	25	27	25	24	27	27
Washington, DC	–	65	**76**	–	65	**72**	64	65	65	63

A double dash means that the city is not present in the sample. For each city in each sample, we computed the difference in the first passage times between the model based on the sample and the model based on the entire network. If this difference was 7 days or larger (6 days was the largest standard deviation), we used bold font in that cell.

Although the data are available for all cities in the samples, we have chosen just a few for illustration. We chose cities that represent major world regions (Athens, Beijing, Bogotá, Cairo, Cape Town, Istanbul, Lagos, London, Moscow, New Delhi, Sao Paulo, Shanghai, and Sydney) that are key entry ports to the United States (Los Angeles, New York, and Washington, DC), or that are among those identified by Guimerà et al. [1] as remote hubs that are unusually weakly connected with the rest of the communities (Barcelona, Detroit, Fairbanks, and St. Petersburg, Russia).

Our simulation results show that for most large and well-connected cities there is little difference in disease dynamics with respect to the sample size. A large expected difference is regional coverage. When the sample size is small some critical cities such as Cape Town or Lagos would be left out of the sample. An example of selection discrepancy between population size vs. flight volume is Washington, DC, which is not a part of the 52 largest city sample but is a part of the 52 largest volume sample. The results also show that larger sample sizes tend to produce smaller FPTs, possibly due to the additional routes through which the disease can travel. Furthermore, when the sample size is large enough, there is no difference between the results from the volume-based and the population-based samples. However, when the sample size is small, air traffic connections to some cities can be inadequately represented, and the disease transmission time can be significantly biased. Most of the discrepancies occur in the small, population-based samples. This is especially true for the large cities that are relatively weakly connected, such as St. Petersburg, Russia, where the FPT is about 130 days for a sample of 52 population-based cities, compared to 94 days in the full network. This difference is more than a month, which is very important for public health preparedness. Similar differences are observed for better-connected cities such as Cape Town (98 vs. 54 days), Bogotá and Washington, DC, although for the latter two the differences are not as dramatic.

These results suggest that ignoring a large number of short connections produces artificial delays in disease transmission. Thus, to avoid bias in disease spread, a reliable sample should include at least the 100–150 cities with the largest flight volume and cities that are well connected. The addition of cities with large population sizes or those in remote locations will improve regional coverage. For example, Fairbanks, Alaska would not be selected in a population-based or a volume-based sample of size n<1000, but given its regional importance, it should be considered for inclusion regardless of the sample size in studies where the Alaskan region is of interest.

The sample of 204 cities adequately represents global disease dynamics while also being reasonably small and providing reasonable regional coverage. (Recall that the sample of 204 cities was formed as the union of the top 100 cities with respect to each of flight volume, population size, betweenness, and degree.) We base our assessment of its ability to represent global disease dynamics on the observation that for our cities of interest it reproduced the FPTs found in the entire list of 2904 cities (within random error). The samples of size 500 and the volume-based sample of size 155 also share this same result. Choosing between these samples may depend on other factors such as size (larger samples require more processing time) and coverage of particular regions.

## Discussion

We have reviewed a number of published global epidemic models and analyzed the global airline transportation network data with respect to its use in epidemic modeling. We have shown that in order to reduce bias in the estimation of global disease dynamics, a network of connected cities should be based on the volume-based core that also includes the most-connected cities. About 150 cities are sufficient for adequate coverage of the major world regions. The results of the simulations suggest that samples based on air traffic volume better describe sample characteristics but can have poor regional coverage and thus need to be complemented by the samples based on population size, betweenness, and clustering characteristics. Next, one could add remote locations for answering questions about specific regions. We have shown through dynamic simulations that a relatively small number of cities (around 200 or 300) can capture enough network information to adequately describe the global spread of a disease such as influenza.

Although the use of 52 cities was justified for the purposes of previous studies [17] and illustrated the role of airline transportation as a major factor in global disease spread, the models that aim to answer more specific questions need to consider a more systematic approach. For example, for practical optimization of the global distribution of antiviral medicine, mathematical models are more likely to consider the role of individual regions or cities in the context of the global spread of disease. If a model is based on a small number of cities, such as in the Rvachev-Longini model, the role of individual cities in the global spread will be underestimated. Incidentally, in a recent paper by Kernéis et al. [17], the authors consider the role of different cities in a global epidemic under different disease characteristics. We would argue that some of their conclusions about the speed of the global spread and the behavior of different city types would change if the authors had considered a more representative set of cities including large metropolitan areas with little traffic as well as smaller but extensively connected cities.

In the present study, we have performed a systematic analysis of network characteristics such as average degree, betweenness, clustering coefficient, and geodesic length, and have shown how these characteristics depend not only on the sample size but also on how the sample is selected. We have provided guidance for the sample size selection when certain network characteristics need to be preserved.

Major network characteristics such as degree, clustering, betweenness, and regional coverage become more biased compared to the entire network as the sample size decreases. The results also show that the bias is smoothly dependent on the population size for all sampling methods and there is no “natural” threshold suggesting the optimal choice of the sample size. For each objective, the “optimal” sample size should be the balance of the scale at which the conclusions are made and the appropriateness of using airline transportation as the basis for human travel flows. One should keep in mind that very small airports can reflect insignificant private traffic flows, while most of the regional transportation is based on ground traffic. Although a large number of network characteristics could be studied with respect to the network samples, we have selected the ones that are clearly defined and were shown to be related to the spread of diseases, innovation, or information. Some measures were inevitably left out. For example, Ghani et al. [18] and Ghani and Garnett [19] demonstrated that network measures such as assortative mixing patterns and population representativeness were strongly affected by the way in which nodes were selected for inclusion in the simulated network. We did not consider the estimation of “mixing” matrices because the actual definition can be ambiguous. Mixing matrices can be defined in terms of flight volume, population size, region, etc. A number of other network measures can be found in Wasserman and Faust [20].

While the field of network analysis is quickly developing, there is still not much literature about the effects of different sampling methods on network characteristics. Most works in network sampling have focused on the problem of estimating how the network measures will change in the sample given a particular sampling scheme such as a snowball, random sampling of nodes and links, or random walk [13], [21], [22]. In our study, we addressed the problem of selecting nodes based on other objectives such as regional coverage and preservation of disease properties rather than just random sampling of nodes or dyads.

The current study has a number of limitations. The analysis is based on the airline network, and its generalizability to other networks such as social or ground transportation might be limited. However, this study is the first attempt to use a systematic approach for the selection of a network sample for dynamic modeling. Although the actual curves and the quantitative results would be different for each specific study, the methodology of selecting a sample based on individual and network characteristics will guide future studies. Another limitation is that in this study the entire network of connected cities is known. Although complete or nearly complete network information is sometimes available in transportation studies, this is usually not the case in social network studies. Nevertheless, having an estimate of which percent of the population the sample represents allows one to extrapolate the network characteristic curves to what they are likely to be in the entire population.
